# Why has under-5 mortality decreased at such different rates in different countries?^☆^

**DOI:** 10.1016/j.jhealeco.2016.03.002

**Published:** 2016-07

**Authors:** Dean T. Jamison, Shane M. Murphy, Martin E. Sandbu

**Affiliations:** a University of California, San Francisco, USA; b University of Lancaster, UK; c Financial Times, UK

**Keywords:** Under-5 mortality, Technical progress, Hierarchical model, Varying coefficients model

## Abstract

Controlling for socioeconomic and geographic factors, under-5 mortality (5q0) in developing countries has been declining at about 2.7% per year, a high rate of ‘technical progress’. This paper adduces theoretical and empirical reasons for rejecting the usual specification of homogeneous technical progress across countries and uses a panel of 95 developing countries for the period 1970–2000 to explore the consequences of heterogeneity. Allowing country-specific rates of technical progress sharply reduces the estimated income elasticity of 5q0 and points to country variation in technical progress as the principal source of the (large) cross-country variation in 5q0 decline. Education levels and physician coverage also contribute and are less affected than income of allowing country variation in technical progress. The paper concludes by decomposing 1970–2000 5q0 decline into its different sources for each country.

## Highlights

• We model 5q0 decline with cross-country heterogeneity in technical progress.
• Estimated income elasticity of 5q0 is reduced from around −0.35 to around −0.12.
• We estimate a high impact of technical progress of around 80% of recent 5q0 decline.
• Geographical context and health systems have strong effects.
• Female education is more important in open than closed economies.

“The rapidity with which the death rate has declined in most of the underdeveloped areas … has been unprecedented. It has never been matched at any time in the now advanced countries … it seems clear that the great reduction of mortality in underdeveloped areas since 1940 has been brought about mainly by the discovery of new methods of disease treatment applicable at reasonable cost [and] by the diffusion of these new methods… The reduction could be rapid because it did not depend on general economic development or social modernization… Though in the literature on public health there is still great lip service paid to the necessity of general economic improvement and community welfare in the control of disease, the truth is that many scourges can be stamped out with none of this…”. (Davis, 1956)

## 1 Introduction

The 20th century differed dramatically from previous history in two critically important domains. First, the rapid economic growth that had begun in the 19th century in the countries of the North Atlantic diffused widely around the globe while continuing in the countries where it originated (Maddison, 1999, DeLong, 2000). Second, human mortality rates plummeted. Again, the changes began in the North Atlantic countries in the 19th century but remained modest until the 20th, during which they accelerated and spread to most of the world (Easterlin, 1996, Easterlin, 1999). Life expectancies typically doubled, entailing major immediate improvements in human welfare, dramatic declines in fertility and, in consequence, transformations of the age structures of populations and their economic environment.

Subsequent to Solow's (1957) assessment of the long-term determinants of income growth in the US, investigators have generated a huge literature on both proximate and deeper-seated determinants of economic growth and on the sources of its variation across countries. Far less attention has been paid to the causes of the mortality transformation, perhaps because its magnitude and rapidity remain less widely known—or are judged less important. Yet, arguably the welfare significance of mortality reduction at least matches that of income growth, and understanding its sources is correspondingly imperative.^1^

Most analysts agree that advances in science and technology underpinned the 20th century transformations of income and mortality levels. Models of economic growth rely heavily on technological progress (defined as changes in total factor productivity) to account for economic growth (Solow, 1957, Boskin and Lau, 2000, Easterly and Levine, 2003). Preston, 1975, Preston, 1980 and Fuchs (1974) provided early quantitative assessments of the central importance of technical progress for life expectancy increases, something anticipated in the Kingsley Davis quote with which this paper begins. While life expectancy and per capita income correlate across countries at any given time, particularly at low income levels, Preston stressed how much average life expectancy has been increasing over time at any given level of income. Some recent econometric works, however, attribute substantial explanatory power to income variations (see Pritchett and Summers, 1996, Filmer and Pritchett, 1999). Yet many middle-income countries today have life expectancies above 75 years with per capita income levels close to what the US had had around 1900, when US life expectancy was only about 49 years. This simple fact supports a deeper investigation of technical progress in health.

In this context, technical progress is more than just changes in the sophistication of drugs, devices and techniques of medicine. It includes improvements in public health provision and private health practices which affect the adoption of the best techniques.^2^ Recent research has either given little emphasis to technical progress—in part simply because much of the research is cross-sectional and therefore ignores developments over time—or it has assumed the rate of technical progress to be constant across countries. But countries differ in how close their health systems come to utilizing the best technology or practice available: the catch-up with the technical frontier may be country-specific. Our purpose in this paper is to model and measure this heterogeneity explicitly.

After introducing our data sources, we explore country-specific technical progress in the decline of under-5 mortality rates or 5q0 (the number of deaths before the fifth birthday per thousand live births). To facilitate estimation, we replace previously used OLS or fixed effects models with hierarchical (or multilevel, varying-effects) models. These models are next used to assess possible correlates of rapid technical progress in mortality decline at the country level. The paper then decomposes improvements in 5q0 into its country-specific constituents, including both country-level determinants explored in previous research and the country-specific rate of technical progress and its determinants.

## 2 Data

Our data set contains observations for 95 low- and middle-income countries for up to seven five-year intervals between 1970 and 2004. A variable value for a specified year is the average for that country of the data available for that and the following 4 years (so GDP in 2000 is the average of GDP from 2000 to 2004). Eighty-seven countries have data on all the variables in our models and we use only these for some of our results. The countries are listed in Web appendix Table D1. The main variables we use are described in Table 1.

We obtain our 5q0 measure from Rajaratnam et al. (2010).^3^ The income variable is real gross domestic product (GDP) per capita in 2000 international dollars from the Penn World Tables (Heston and Summers, 1996, Summers and Heston, 1991), with some missing data interpolated.^4^ The educational measure is the average number of years of schooling for women aged between 25 and 34 (Lutz et al., 2007). We also use the number of physicians per 100,000 people, taken from the United Nations (1950–2009) as collected in Banks (2010).

We use a set of geographical and policy variables constructed by Harvard University's Center for International Development to generate improved models of the determinants of economic growth rates, in order to see if they also predict country-specific rates of mortality decline. Gallup et al. (1999) measure the percentage of a country's population living in the geographical tropics (our variable TROPICS) and within 100 km of a coast or navigable waterway (COASTAL). Economic openness (OPENNESS) is the (time-invariant) percentage of years between 1965 and 2003 that the country's economy was considered open estimated in Wacziarg and Welch (2007), which builds on similar work by Sachs and Warner (1995). We also include a health policy measure as a potential determinant of technical progress. The coverage of a child's third immunization with the diphtheria, pertussis, and tetanus vaccine (DPT3) in 1986 (Lim et al., 2008) provided a natural indicator of the extent to which a country's health services are early adopters of powerful mortality reducing technologies.

**Fig. 1:**
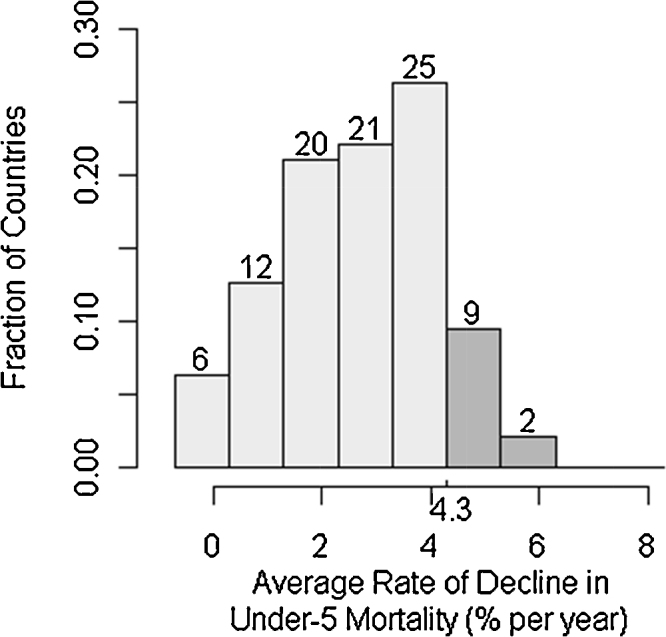
Rates of decline in under-5 mortality, 95 countries, 1970–2000. Note: Values are grouped in 1% increments above and below 4.3%, the rate required to reach MDG-4.

Table 1 shows that between 1970 and 2000 per capita income and the average female education level both roughly doubled. The average 5q0 was 143 in 1970 and 62 in 2000. The mean decline across all countries over that period was 3.4% per annum. It should be noted that the cross-country *variation* in the rate of 5q0 decline is itself quantitatively important. Fig. 1 displays its distribution. As the histogram shows, there is a dramatic spread across countries. Eight countries reduced 5q0 by less than 0.5% per year, while 11 countries had an annual rate of reduction greater than the 4.3% required to meet Millennium Development Goal 4 (MDG-4), which is to reduce 5q0 by two-thirds between 1990 and 2015.

## 3 The importance of heterogeneous time trends

We think that in some important sense most scientific and technical knowledge is globally available.^5^ However, we relax the standard assumption that the rate of take-up or diffusion of available technology and know-how is the same in each country. The spirit of our approach echoes Lee et al. (1997), who in the context of modeling economic growth allowed not only for country-specific effects on income *levels* (time-invariant fixed effects), but also for country-specific *rates* of productivity growth. This paper goes beyond previous work on mortality decline by similarly modeling different rates of technical progress across countries. It also models some possible *determinants* of why the rate of technical progress varies.^6^ We view this exploration of potential determinants as suggestive and far from definitive. That said, a number of factors do appear robustly related to the (very large) cross-country variation of technical progress in mortality decline.^7^

We now present models to incorporate our desired parameter heterogeneity, then show diagnostic results to establish the importance of doing so before moving on to our main substantive results.

### 3.1 Models

Standard econometric analyses of the determinants of cross-country variation in health outcomes consist of multivariate cross-sectional (see Filmer and Pritchett, 1999) or panel regression models (Pritchett and Summers, 1996).^8^ Since our interest is in investigating the effect of technical progress on health outcomes over time, our focus will be on panel models. Consider, then, the following model of the determinants of the natural logarithm of under-5 mortality:

(1) $$\text{ln}\;5\text{q}0_{it}=\beta_{0}+\beta_{1}\text{TIME}+\beta_{2}{\text{ ln GDPPC}}_{it}+\beta_{3}{\text{EDFEMALE}}_{it}+\beta_{4}{\text{ ln DOCSPC}}_{it}+\varepsilon_{it}\text{,}$$

where, TIME is the year *t*, other variables are defined in Table 1, and *ɛ*_*it*_ is a random, i.i.d., normally distributed disturbance. A common extension is to allow the intercept (*β*_0_) to vary across countries, leading to:

(2) $$\text{ln}\;5\text{q}0_{it}=\beta_{0i}+\beta_{1}\text{TIME}+\beta_{2}{\text{ ln GDPPC}}_{it}+\beta_{3}{\text{EDFEMALE}}_{it}+\beta_{4}{\text{ ln DOCSPC}}_{it}+\delta_{it}\text{,}$$

where, *β*_0*i*_ is a country-specific intercept and *δ*_*it*_ is a random, i.i.d., normally distributed disturbance. Neither (1) nor (2) allow for heterogeneity in the variable coefficients, and assume in particular that the (conditional) trend change in the health outcome over time (*β*_1_) is the same in all countries.

As we have explained, there is little reason to assume *ex ante* that countries are identical in their abilities to avail themselves of better knowledge and improved techniques for reducing mortality. Suppose that instead of Eq. (2), the true model is:

(3) $$\text{ln}\;5\text{q}0_{it}=\beta_{0i}+\beta_{1i}\text{TIME}+\beta_{2}{\text{ ln GDPPC}}_{it}+\beta_{3}{\text{EDFEMALE}}_{it}+\beta_{4}{\text{ ln DOCSPC}}_{it}+\nu_{it}\text{,}$$

where, *β*_1*i*_ is a country-specific time trend and *ν*_*it*_ is a random, i.i.d., normally distributed disturbance. If so, important problems arise from imposing Eq. (2) to estimate a relationship that in reality follows Eq. (3). If the true model is Eq. (3), then the error term in Eq. (2) can be rewritten as:

(4) $$\delta_{it}=\left(\beta_{1i}-\beta_{1}\right)\text{TIME}+\nu_{it}$$

In other words, the error term in Eq. (2) will both be autocorrelated and correlated with the regressors (because of the presence of the time trend). This creates the risk of biased estimates of the true coefficients, which, we argue below, has in fact occurred in previous work finding income to be a strong determinant of mortality. In addition, using Eq. (2) to estimate Eq. (3) leaves us ignorant about cross-country variation in technical progress over time.

How serious is this risk? Econometric theory shows that in the presence of serially correlated variables (those that change gradually over time, which the determinants of mortality do) imposing coefficient homogeneity leads to systematically biased estimates.^9^ Several empirical studies in other areas suggest the problem can be quantitatively significant.^10^ We propose that such bias afflicts the extant literature on cross-national determinants of health outcomes, and that it is necessary to allow for coefficient heterogeneity when estimating the strength of these determinants.

In our estimations below, we extend Eq. (3) with the following specifications for *β*_0*i*_ and *β*_1*i*_:

(5a) $$\beta_{0i}=\gamma_{00}+\mu_{0i}$$

(5b) $$\beta_{1i}=\gamma_{10}+\mu_{1i}$$

These equations decompose the intercept and the time trend in the main equation into a country-invariant and a country-specific component. The country-specific components *μ*_0*i*_ and *μ*_1*i*_ are assumed to be normally distributed, zero-mean random variables which are independent of the unexplained error term in the overall equation (formally $\text{Cov}\left(\mu_{0i},\varepsilon_{0i}\right)=0$ and $\text{Cov}\left(\mu_{1i},\varepsilon_{1i}\right)=0$). This simple specification preserves the standard assumption of a common health production function across countries except for country-specific level shifts or time trends. With the error structure given in Eqs. (5a) and (5b), it is equivalent to a hierarchical linear model, which can be written in the following single-equation form:

(6) $$\ln\;5\text{q}0_{it}=\gamma_{00}+\gamma_{10}\text{TIME}+\beta_{2}{\text{ ln GDPPC}}_{it}+\beta_{3}{\text{EDFEMALE}}_{it}+\beta_{4}{\text{ ln DOCSPC}}_{it}+\left(\mu_{0i}+\mu_{1i}{\text{TIME}}_{t}+\varepsilon_{it}\right)$$

Below, we use a restricted maximum-likelihood algorithm to estimate this model, and report results for more complex error structures in the Web appendix.

To assess how much of the cross-country coefficient variation can be explained by country characteristics we also explore alternative level-2 models which include the time-invariant variables mentioned above as possible determinants of the country-specific coefficients in the level-1 model:

(7a) $$\beta_{0i}=\gamma_{00}+\gamma_{01}{\text{TROPICS}}_{i}+\gamma_{02}{\text{COASTAL}}_{i}+\gamma_{03}{\text{IMMUNIZATION}}_{i}+\gamma_{04}{\text{OPENNESS}}_{i}+\mu_{0i}$$

(7b) $$\beta_{1i}=\gamma_{10}+\gamma_{11}{\text{TROPICS}}_{i}+\gamma_{12}{\text{COASTAL}}_{i}+\gamma_{13}{\text{IMMUNIZATION}}_{i}+\gamma_{14}{\text{OPENNESS}}_{i}+\mu_{1i}$$

In the diagnostic results reported in the next section, we estimate a simple version of Eqs. (7a) and (7b) that only includes immunization coverage as a level-2 predictor. Later we present full results.

### 3.2 Diagnostic results

Table 2 reports our estimation of Eqs. (1), (2), (6), and (7). The generic model in column A imposes a common intercept and time trend on all countries. The model in column B allows for a varying country-specific intercept shift but retains the constraint of a common time slope. This benchmark model is very similar to the specification in Pritchett and Summers (1996).^11^ As we relax the homogeneity assumption in columns C and D we first let the time trend vary as well and then include a level-2 determinant (immunization) for both the intercept and the time trend. In order to estimate the different models, we use the restricted maximum-likelihood procedure known as hierarchical linear modeling or HLM.^12^

The estimates in column B are very close to those found in the previous literature, *e.g.*, Pritchett and Summers (1996). In particular, the estimated −0.26 elasticity of 5q0 with respect to income is close to their estimates between −0.2 and −0.4 and implies that a 10% increase in real per capita GDP would reduce 5q0 by 2.6%. Given economic growth rates in the 1970–2000 period, this would mean GDP growth and technical progress in mortality reduction contributed about the same to observed 5q0 declines.

As discussed above, however, the complex error structure makes least squares methods used in the earlier literature biased. The reason for this is that models without a randomly varying time slope (columns A and B) are constrained versions of the equivalent model that allows this variation. For example, Eq. (2) (estimated in column B) is a restricted version of Eq. (6) (column C) which constrains the variance of the country-specific slope and its covariance with the intercept to be zero. Statistical tests of whether this restriction is valid overwhelmingly indicate that it is not.^13^

What is of interest is how the coefficient on income changes when the assumption of identical technical progress across countries is relaxed, as the data indicate it should be. Column C shows that the income effect is then much smaller than what has been found in cross-country regressions. A 5q0-elasticity with respect to income of −0.122 implies that a doubling of GDP, *ceteris paribus*, is associated with a fall in the under-5 mortality rate of only about 8.1% (*e*^−0.122(ln(2))^ ≈ 0.919). This suggests that for reducing under-5 mortality, purely growth-oriented policies may not be particularly effective. Instead it may be much more important to understand the cross-country differences in adoption of low-cost, life-saving technologies.^14^ Easterly (1999) found a broadly similar pattern of findings on the importance of income for other social indicators. (In Web appendix C, we show that this low estimate of the income elasticity under heterogeneous time-slopes is robust to a number of other estimation choices.)

The fact that the income coefficient falls when country-specific time trends are allowed has an important substantive interpretation. It means, on the one hand, that much of the strong effect of income on mortality outcomes found in previous work simply captures the pattern that countries that have grown rich fast have also cut mortality fast. On the other hand, it means that there is little effect of *changes* in incomes around long-term trends—accelerations, slowdowns and fluctuations in GDP has little bearing on mortality outcomes. That supports our general conclusion that health depends more on the accumulation of technology and know-how over time than on the availability of resources.

*To summarize*: There are no *ex ante* reasons to believe that rates of technical progress are the same for all countries. Econometric theory shows that imposing slope homogeneity when slopes in reality are country-specific leads to inconsistent estimates with conventional methods when the variables are serially correlated. And a cursory examination of data on under-5 mortality rates and their determinants reveals that this inconsistency may lead to a serious misunderstanding of the relationships between per capita income, technical progress and health outcomes. In the next section, we employ a varying coefficients model to take seriously the possibility of country-specific rates of technical progress, and investigate the determinants of these country differences.

## 4 Determinants of under-5 mortality

The previous section provided an overview of the strong intuitive, theoretical and empirical reasons for relaxing the assumption of time trend homogeneity in the econometric analysis of cross-country variations in health outcomes.^15^ We now proceed to analyze more detailed models of the determinants of child mortality rates when that assumption is relaxed. These models are in other respects similar to earlier approaches.

### 4.1 Selection of models

We make three important modeling choices, relating to (a) the functional form of our estimating equations; (b) the choice to model heterogeneous coefficients as (conditionally) randomly distributed rather than a dummy variable approach; and (c) the choice to retain the assumption of homogeneous coefficients for variables other than time. We discuss each of these in turn.

#### 4.1.1 Functional form of the estimating equation

Using the log of under-5 mortality rates on the left-hand side (instead of raw levels) allows comparison of our results with extant findings, and has a theoretical and practical advantage that the coefficients are (conditional) elasticities or semi-elasticities of 5q0 with respect to a predictor of interest. Estimating elasticities reflects the plausible idea that it should take more resources to reduce 5q0 by a given absolute amount the lower is its current level.

#### 4.1.2 Varying coefficients or indicator variables

For clarity we follow the vocabulary described in Gelman and Hill (2007). When country specific coefficients are estimated using a hierarchical linear model as in Eq. (6) we describe coefficients as varying and we describe our models as varying effect models.

A varying coefficients approach has two main advantages over fixed effects. First, in these short time series (a maximum of seven time periods per country) estimating coefficients on two interaction terms per country (for the intercept and for the time slope) would use twice as many degrees of freedom as we have countries. Second, we are interested not just in allowing the intercept and the time slope to vary across countries, but also to model this variation as a function of time-invariant country characteristics. This we cannot do directly with an indicator variable approach, since the coefficient on an indicator interacted with time will pick up all the time-invariant cross-country variation.

#### 4.1.3 Remaining coefficient homogeneity

If we think that coefficients vary across countries, why do we content ourselves with relaxing the homogeneity assumption for the time coefficient, without allowing country-specific estimates for the other determinants? We do report results from such estimations in Web appendix B and find our main results are robust allowing country-specific estimates for income, female education, or doctors per capita. However, as a first step towards relaxing the assumption of slope homogeneity, country-specific rates of technical progress is a natural and parsimonious approach, and it is conceptually very close to the conventional practice of letting intercepts vary. The standard specification, with a log transformation of q50 and country level effects, implies that countries differ by a fixed multiplicative productivity shift (additive in the log form) in how well they make use of a given set of inputs to achieve mortality outcomes. This time trend simply means that this productivity shift changes over time. Our approach, then, is to let productivity levels, assumed to differ between countries in the literature, also *change* at different speeds. This is hardly a great conceptual leap. We therefore think it is useful to begin by concentrating on this conceptually small but empirically consequential modification to current practice. Moreover, Web appendix B shows that allowing the time trend to vary has much greater implications for the estimated effects of the other variables (and contributes more to model fit) than does country-specific heterogeneity in the other coefficients.

### 4.2 Determinants of the varying effects

The estimates for our benchmark model are reported in Table 2 (model C). Other things equal, the estimated time trend means that under-5 mortality rate decreases by 2.7% per year on average across countries. This impact of technical progress dwarfs the effects of the other variables. The elasticity of 5q0 with respect to income is −0.122. A 10% (or $328 at the sample mean) increase in income is associated with a 1.2% fall in under-5 mortality, or about 1.1 fewer deaths per thousand births at the sample mean. We estimate that one additional year of female education is associated with about a 3.6% fall in under-5 mortality (or about 3.1 fewer deaths per thousand at the sample mean). A 10% increase in the number of physicians per capita would reduce under-5 mortality by 1.1%. These effects are all small compared to technical progress.

Table 3 reports models that introduce the geographical and public health variables to see if they contribute to explaining cross-country differences. Model E includes them in the level-2 model for the intercept. A completely tropical country (TROPICS = 1) has, on average, a 31% higher under-5 mortality rate than a non-tropical one. A country whose entire population lies within 100 km of the coast or a navigable river (COASTAL = 1) has, on average, a 50% lower under-5 mortality rate than a completely landlocked one. A country whose entire population had received DPT3 vaccination has, on average, a 64% lower under-5 mortality rate than one with no DPT3 penetration. These results are, of course, hard to interpret conclusively. They could reflect the different disease environments or the relatively low productivity of tropical agriculture. Or there could be omitted variable bias if, for example, tropical countries have less developed health care infrastructure for reasons not related to the climate itself. As for immunization rates, they are correlated with many measures of development, so we discuss in Web appendix C whether other development variables are more appropriate.

### 4.3 Determinants of technical progress

We see in columns F–I of Table 3 that the coefficients in the level-2 model of the time slope are both statistically significant and quantitatively important. Model F estimates, for example, imply that an average temperate, coastal country (TROPICS = 0; COASTAL = 1) reduces its 5q0 by 4.4% per year, about three times as fast as an average tropical and landlocked country (TROPICS = 1; COASTAL = 0), whose rate is 1.4% per year. Models G and H show that economic openness also contributes greatly to mortality reduction: the economically most open countries (OPENNESS = 1) on average have a rate of technical progress in 5q0 outcomes that is around 2 percentage points per year faster than economically closed, but otherwise similar countries. In 25 years, this substantial differential accumulates to an additional 39% reduction in 5q0. Countries that are not open to the world economic system miss out not only on gains from trade and specialization in production, but also on information flows and the benefits of technological diffusion in health.^16^

Finally, in model I we also included DPT3-86 as a determinant of the TIME coefficient, and found that countries with greater vaccine coverage, too, have significantly faster mortality declines, independently of income, educational attainment, or doctors per capita. Specifically, a 10 percentage point increase in 1986 vaccine coverage is associated with 0.19 percentage point faster annual rate of mortality decline. Our interpretation is that DPT3 coverage proxies for a health system's orientation toward early introduction of a range of high priority interventions.

Even after adding these country characteristics to explain country-specific technical progress, large cross-country variations in the time slope remain, captured by the random term *μ*_1*i*_ of Eq. (6). For many countries, this unexplained part of the country-specific time trend is of comparable magnitude to the effects of the geographic, openness and vaccination variables. The standard deviation of *μ*_1*i*_ is between 0.010 and 0.015, or more than one percentage point per year in the rate of technical progress. That is substantial given an average overall time trend (calculated with the determinants at their mean values) of 2.6–2.9% per year. The importance of a 1.5 percentage point better (more negative) rate of technical progress is illustrated by a simple calculation: After 25 years, that one-standard deviation improvement makes 5q0 a full 31% less than what it would be in an otherwise identical country.

## 5 Decomposing the decline in 5q0

We highlight three main empirical findings from the previous sections. First, the raw annual rate of mortality decline varies enormously across countries, with a range of −0.5 to 8% per year around a mean of about 3.4% per year. Second, when rates of improvement over time, or technical progress in mortality reduction, are modeled explicitly as varying across countries, the estimated effect of income on 5q0 outcomes is substantially reduced, but this is less true for the impact of education and doctor coverage. Third, there are clear correlations between technical progress and geographic characteristics, as well as between technical progress and economic and health system variables.

In this section we add one further insight. Just as the economic growth literature reports decompositions of growth into elements associated with increased levels of inputs and productivity growth, so, too, 5q0 declines can be decomposed into the contribution of different factors. For each country and for the sample as a whole we identify the estimated contributions to 5q0 decline due to each of four components: the decline due to changes in income levels, education levels, doctor coverage, and technical progress.

5q0 in low- and middle-income countries declined dramatically in the period 1970–2000, from 143 per thousand to 63. The average per capita income increased by 78% (from $2300 to $4100) and the average length of women's education increased from 3.4 to 7.5 years. Doctor coverage trebled from 4.4 to 13.4 doctors per 1000 people. How much did each of these factors contribute to the total 5q0 decline? And how much of the decline remains unaccounted for by these factors and, by convention, can be attributed to technical progress? The answers to these questions are country-specific both because the input changes are country-specific and because the calculated rate of technical progress is.

**Fig. 2:**
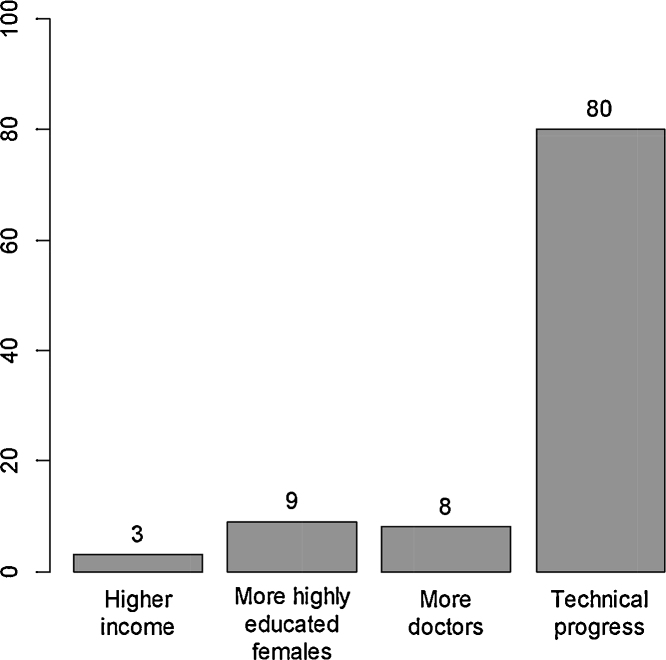
Factors accounting for decline in under-5 mortality, all low- and middle-income countries, 1970–2000. Note: Under-5 mortality (5q0) averaged across all countries declined from 143 per thousand in 1970 to 62 per thousand in 2000. The average country-specific rate of decline was 3.4% per year. This graph shows the contribution of selected policy-elated determinants of this decline. Decline associated with technical progress is broken down into geographic, DPT3 immunization coverage, and unexplained country effects in Web appendix D.

We use the estimates from the fullest model, reported in column J of Table 3, for the decomposition. For each country, we measure the change in each of the inputs, apply the estimated effects of these inputs, and add the total country-specific technical progress over the period (30*β*_1*i*_) to calculate a predicted change in the log of 5q0. The decomposition then divides the predicted effect on 5q0 of each factor by the total predicted 5q0 decline. At sample averages we attribute 3% of the 5q0 decline to increases in per capita income, 8% to improvements in female education, 9% to the increased number of physicians and 80% to technical progress (Fig. 2). While technical progress explains 80% of mortality improvements across countries, its importance varies widely across countries—from 54% in China (whose rate of 5q0 decline is 3.5% per year) to 82% in India (whose pace is 2.9%) to 83% in Mexico (whose pace is 4.8%). This wide variation in realized technical progress provides most of the answer to the question posed in the title of this paper: “Why has under-5 mortality declined at such different rates in different countries?” Web appendix Table D2 reports decompositions by country.

## 6 Conclusion

The 20th century witnessed huge and unprecedented declines in mortality rates at all ages and in most parts of the world. Understanding the sources of these changes is important not only for understanding one of the defining events of world history but, also, to devise policies to address the needs of the quarter of the world's population whose mortality rates remain far higher than those of the rest of humanity.

Several approaches shed light on the sources of mortality decline. Epidemiologists and demographers have carefully tracked specific communities for many years to assess what causes mortality decline, and for what reasons. An interesting example of this approach found, in rural Senegal, that much of the rapid mortality decline there could be traced to the introduction of interventions addressing specific conditions (Pison et al., 1993). Another approach is historical. Easterlin, 1996, Easterlin, 1999, for example, examined the interplay of economic growth, urbanization and mortality in 19th and 20th century Europe. He found little correlation between the timing of periods of economic growth and mortality decline and concluded that income growth in the 19th century probably had a real but modest impact on reducing mortality through its influence on food availability and environmental conditions. Fogel (1997) has also stressed the importance of increases in food availability during this period. These positive factors were partially offset by increased infectious disease transmission resulting from urbanization. Easterlin concludes that 20th century mortality decline, which was much more rapid than that of the 19th century, had its origin in technical progress, and Powles (2001) has pointed to the importance and nature of the institutional changes required to translate technical change and economic improvements into mortality reduction.

Increasingly good time series data have become available on country-specific demographic and economic conditions for the period from around 1960. These data have allowed statistical assessment of relations among income, education, technical progress and mortality, a line of work initiated by Preston, 1975, Preston, 1980. This paper adds to that literature by continuing to explore of the role of geographical variables (work begun by Bloom et al., 1999) and, more importantly, by allowing for heterogeneity across countries in the rate of technical progress in mortality decline. We find that there is high variation across countries in the rate of improvement over time and that taking account of that variation substantially reduces the estimated effect of income on health. Even in a period of rapid economic growth income changes can account for only a modest fraction of the changes in under-5 mortality in most countries. Variations in technical progress and (to a lesser extent) educational improvements are far more important in explaining why under-5 mortality has declined so much, and at such different rates in different countries.

Global collective efforts have played increasingly substantial roles in the period covered by this study. Most of these lie in the public sector, including well-funded programs to expand immunization coverage, to treat childhood diarrhea and pneumonia, and to prevent and treat malaria. Best practices spread, but unevenly because of both variations across countries in funding received and variation in the learning afforded by participation in the technical governance of the programs. Variable participation in private sector clinical trials may also have contributed to differential uptake of innovation. We would speculate that the World Health Organization as well as development assistance agencies played a critical role in accelerating diffusion of life-saving innovations around the world. At the same time variable participation in WHO's work and uptake of its recommendations may have contributed to high variance in progress around a high mean.

Drawing on Mosk and Johansson's (1986) assessment of the interplay between income and mortality in Japan, it may be an instructive simplification to categorize mortality history into 3 or 4 epochs. Epoch I, extending up to the late 18th century, was a period of ups and downs in mortality rates unaccompanied by any upward trend. Epoch II, in the 19th century, witnessed slow but real mortality reductions among the North Atlantic countries that resulted from improved diets and other consequences of income growth, but that were partially counterbalanced by the adverse effects of urbanization. Epoch III, in the 20th century, was a period of very rapid mortality decline in much of the world that was based on the generation and diffusion of inexpensively applied new and existing knowledge and specific technologies embodying that knowledge. The *Lancet* Commission on Investing in Health (Jamison et al., 2013) pointed to an Epoch IV, in the first third of the 21st century, involving convergence of all countries’ mortality rates to the levels technology has now made possible even at low levels of income.

## Figures and Tables

**Table 1:** Variables in the analysis: definitions, means and standard deviations .

Variable	Definition	All Years	1970	1985	2000
GDPPC	GDP per capita in 1990 international dollars, Penn	3141	2346	2881	4096
World Tables (Heston et al., 2009)	(2680)	(2075)	(2267)	(3493)

lnGDPPC	Natural logarithm of GDPPC	7.697	7.451	7.632	7.935
	(0.87)	(0.79)	(0.86)	(0.93)

5q0	Under-5 death rate or probability of dying prior to 5th	97.87	142.9	99.21	62.2
birthday (per 1000 live births), Rajaratnam et al. (2010)	(75.32)	(82.42)	(72.37)	(52.66)

ln5q0	Natural logarithm of 5q0	4.221	4.794	4.285	3.643
	(0.93)	(0.66)	(0.86)	(1.00)

EDFEMALE	Average number of years of education in females aged 25–34, Lutz et al. (2007)	5.58	3.424	5.32	7.465
(3.31)	(2.59)	(2.98)	(3.18)

DOCSPC	Number of Physicians per 100,000 people—United Nations (1970–2004) and Banks (2010)	874.9	435.4	678.1	1341
(1119)	(534)	(820)	(1331)

lnDOCSPC	Natural logarithm of DOCSPC	5.842	5.282	5.651	6.483
(1.52)	(1.40)	(1.50)	(1.38)

TROPICS	Fraction of population living in the geographical Tropics, Gallup et al. (1999)	0.391	0.359	0.348	0.444
(0.47)	(0.47)	(0.46)	(0.48)

COASTAL	Fraction of population living within 100 km of the coast, Gallup et al. (1999)	0.474	0.490	0.471	0.480
(0.37)	(0.37)	(0.37)	(0.38)

OPENNESS	Fraction of years during 1965-2003 economy is open, Sachs and Warner (1995) and Wacziarg and Welch (2007)	0.324	0.347	0.334	0.304
(0.29)	(0.30)	(0.30)	(0.29)

DPT3-86	DPT3 Vaccination Coverage in 1986, Lim et al. (2008)	0.656	0.644	0.644	0.674
(0.20)	(0.21)	(0.21)	(0.19)

	Number of Observations	527	68	71	85

*Note*: Entries in the table are the means for the indicated time periods with the standard deviation in parentheses below.

**Table 2:** Determinants of under-5 mortality: diagnostic results.

Model	A	B	C	D
Equation	(1)	(2)	(6)	(6,7)
Coefficient estimates				
Intercept (constant across countries)	8.452	.	.	.
(−44.05)			
TIME (constant across countries)	−0.013	−0.021	.	.
(−6.33)	(−13.88)		
lnGDPPC	−0.345	−0.257	−0.122	−0.095
	(−11.08)	(−8.06)	(−4.26)	(3.33)
EDFEMALE	−0.121	−0.074	−0.036	−0.027
	(−11.47)	(−6.87)	(−3.49)	(−2.60)
lnDOCSPC	−0.101	−0.105	−0.112	−0.123
	(−4.04)	(−4.05)	(−6.54)	(7.37)
Level-2 model: determinants of TIME coefficient				
TIME (constant component)—γ_10_		.	−0.027	−0.001
		(−13.63)	(0.25)
SD of variance component of TIME–SD( μ_1i_)			0.015	0.013
DTP3-86				−0.40
			(5.49)
Level-2 Model: Determinants of the Intercept				
Intercept (constant component)—γ_100_		8.135	7.388	7.049
	(−30.05)	(24.92)	(26.60)
SD of variance component of Intercept–SD( μ_0i_)		0.360	0.517	0.53
DTP3-86				−0.38
			(1.21)
Estimation Statistics				
*N*	573	573	573	573
Countries	95	95	95	95
RMSE	0.425	0.152	0.06	0.06
*R*^2^	0.799	0.974	0.996	0.996
AIC	658	−58	−554	−559

*Note*: Robust *t*-statistics in parentheses under the point estimates. The dependent variable is logged 5q0. Unbalanced panel using up to 7 time periods for each country.

**Table 3:** Determinants of log under-5 mortality: the effects of income, education, physician coverage, geography, and technical progress.

	E	F	G	H	I	J
Level-1 model						
Mean intercept effect	6.452	6.377	6.389	6.447	6.463	6.442
Mean TIME slope	−0.028	−0.029	−0.029	−0.028	−0.026	−0.027
lnGDPPC	−0.113	−0.086	−0.106	−0.106	−0.107	−0.107
(−3.94)	(−3.02)	(−3.52)	(−3.53)	(−3.55)	(−3.64)
EDFEMALE	−0.032	−0.03	−0.016	−0.016	−0.129	0.028
(−3.09)	(−3)	(−1.58)	(−1.58)	(−1.25)	(2.21)
lnDOCSPC	−0.11	−0.114	−0.077	−0.077	−0.080	−0.087
(−6.47)	(−6.82)	(−4.45)	(−4.45)	(−4.64)	(−5.19)
EDFEMALE × OPENNESS	.	.	.	.	.	−0.185
					(−5.44)
Level-2 model: determinants of TIME coefficient						
TIME (constant component)–γ_10_	−0.028	−0.026	−0.025	−0.025	−0.014	−0.018
(−13.89)	(−8.7)	(−8.56)	(−8.68)	(−2.52)	(−3.14)
SD of variance component of TIME–SD( μ_1i_)	0.015	0.011	0.011	0.012	0.010	0.011
TROPICS	.	0.012	0.013	0.013	0.010	0.010
	(4.24)	(4.76)	(4.78)	(3.48)	(3.20)
COASTAL	.	−0.018	−0.013	−0.013	−0.011	−0.011
	(−4.94)	(−3.75)	(−3.79)	(−3.12)	(−2.84)
OPENNESS	.	.	−0.019	−0.02	−0.018	0.011
		(−4.09)	(−5.36)	(−3.98)	(1.50)
DPT3-86	.	.	.	.	−0.017	−0.019
				(−2.41)	(−2.56)
Level-2 model: determinants of intercept						
Intercept (constant component)–γ_00_	7.447	7.23	7.269	7.009	7.009	6.889
(28.46)	(27.26)	(26.5)	(23.9)	(22.39)	(22.37)
SD of variance component of intercept–SD( μ_0i_)	0.517	0.452	0.454	0.450	0.450	0.448
TROPICS	0.268	−0.009	0.067	0.137	0.137	0.135
(2.69)	(−0.07)	(0.55)	(1.11)	(1.11)	(1.10)
COASTAL	−0.695	−0.273	−0.333	−0.393	−0.393	−0.375
(−6.1)	(−1.96)	(−2.22)	(−2.61)	(−2.61)	(−2.50)
OPENNESS	.	.	−0.047	−0.0721	−0.072	0.426
		(−0.25)	(−0.38)	(−0.38)	(2.03)
DPT3-86	−1.014	−1.024	.	−0.824	−0.824	−0.745
(−3.92)	(−3.99)		(−2.687)	(−2.69)	(−2.44)
Estimation statistics						
*N*	573	573	527	527	527	527
Countries	95	95	87	87	87	87
RMSE	0.06	0.06	0.057	0.057	0.057	0.055
*R*^2^	0.996	0.996	0.996	0.996	0.996	0.997
AIC	−562	−577	−581	−584	−576	−597

*Note*: Robust *t*-statistic in parenthesis under estimate, dependent variable is logged 5q0. All models assume heterogeneity both in level and in time.

## Footnotes

1 Easterlin (1996) and Crafts (2000) placed an emphasis on mortality transformation that is comparable to their emphasis on economic growth in their retrospectives on the unprecedented changes in the human condition during the 20th century, whereas DeLong (2000), for example, places far more exclusive emphasis on the growth of income (and on the availability of altogether new material goods). When reasonable estimates of the dollar value of mortality reduction are added to the value of material output growth, however, 20–50% of the growth in total economic welfare has been attributed to mortality reductions for different countries in different eras. Usher (1973) provided the first such estimates; Mokyr and Stein (1997) provided estimates for high-income countries in the late 19th and early 20th century; Nordhaus (2003) provided recent estimates for the US; and Jamison et al. (2013) concluded mortality decline's annual value in low- and middle-income countries to have been worth over 1% of GNI in the period 1990–2011.

2 Adams et al. (2003) used micro-data to more closely study the causal path between socio-economic variables and improved health. Cutler et al. (2006) provided an excellent recent review of the determinants of health.

3 The correlation coefficients by year between this series, from UNICEF (2009) and from the 1999 World Development Indicators range from 0.994 to 0.998 depending on the year (authors’ calculations). Hill and Amouzou (2006) provided a thoughtful discussion of the difficulties in measuring 5q0, and how those can be addressed.

4 In Web appendix A we test our model using different data sets for income (measured both by PPP and by the “Atlas Method”), education, physicians per capita, and country samples and find our results to be consistent across these choices of variables.

5 Patent restrictions on products relevant to health do of course entail availability of patented commodities only at prices well above the marginal cost of production and distribution. With few exceptions, however, patents are unimportant barriers for access to products capable of influencing major mortality decline.

6 Fuchs (1980) observed some time ago that different groups of countries differ in how their mortality changes relate both to income change and to technical progress. This paper can be viewed as drawing out the consequences of Fuchs's observation at the level of countries rather than groups of countries.

7 In related work we have undertaken a parallel analysis of the determinants of country-specific variation in the rate of change of total factor productivity for explaining differences in economic growth rates across countries (Jamison et al., 2005). This specification is also used in Jamison et al. (2007).

8 In a paper dealing with infant mortality decline in Sweden, the UK and the US, Bishai (1995) has developed a cointegration approach for dealing with the long time series available for those countries.

9 Robertson and Symons (1992) constructed dynamic panels with a serially correlated regressor (assumed to be the true determinant of the dependent variable) and a lagged dependent variable (with no causal effect). If the regressor is a random walk, the coefficient on the lagged variable is biased upwards and the coefficient on the true determinant is biased downwards when coefficient homogeneity is erroneously imposed. Pesaran and Smith (1995) found that incorrectly imposing slope homogeneity yields inconsistent estimates of the true average slopes in fixed or random effects panel regressions with serially correlated regressors.

10 Lee et al., 1997, Lee et al., 1998 showed that the slow convergence of per capita incomes between countries observed in economic growth research is exaggerated by assuming an identical rate of technology growth across countries.

11 One difference is that they model the intercept shift as a fixed effect; we model it as a random or variable effect. We discuss this modeling choice in some detail below; the difference it makes to the other coefficient estimates is negligible.

12 For the econometric details of the estimation method, see Bryk and Raudenbusch (1992) and Kreft and De Leeuw (1998). We estimate the models in R (version 2.10.0) using restricted maximum likelihood estimation with the command lmer from the package lme4 (Bates et al., 2008) for the hierarchical models, and with the command lm from the base package for the pooled, fixed effects, and difference models reported in the Web appendix.

13 The bottom row of Table 2 gives our preferred indicator, the Akaike information criterion (AIC). The AIC is calculated as *AIC* = 2*k* − 2 ln *L*, where *k* is the number of parameters in the model and *L* is the maximized value of the likelihood function (so the AIC penalizes a model that includes more parameters to avoid overfitting). A smaller (or more negative) AIC then reflects a specification that is more likely to be the best representation of the true model. On this criterion, the models with country-specific technical progress over time are vastly preferable to the more constrained models. The difference between two models’ AIC is their logged likelihood ratio adjusted for the different number of parameters estimated, so this number can be used for a likelihood ratio test between two chosen models. Between models B and C (C estimates two more parameters than B) the test statistics (554 – 58 + 2) = 498 is well above critical values at conventional significance thresholds. Again, this suggests it is invalid to constrain the time trend to be the same for all countries.

14 Much empirical evidence points, however, to a strong within-country correlation of income and health (e.g., Wagstaff et al., 2004). Deaton (2001) pointed to one interpretation, which is that an individual's health may be affected by her standing relative to a salient reference group (rather than her income level *per se*), which in this case would be the country. This is logically consistent with a much weaker relationship between income and health across countries or in a single country over time.

15 Oeppen (2006) independently applied models closely related to ours to assess (for a smaller group of countries over a much longer time frame) country-specific elasticities of mortality with respect to income levels and country-specific rates of technical progress (although he uses different terminology).

16 An alternative view of the important of openness is that it influences the gains to education rather than technical progress over time. We test this in model J, in which the education variable is interacted with openness in the level-1 equation. The results bear out the intuition: the effect of openness on the time slope becomes insignificant, while the level-1 coefficient on the interacted variable is large and significant. Consistent with a model of economics of education going back to Schultz (1961), our model finds that the positive effect of education is greater in environments having greater economic openness.
